# The Association between Admission Serum Phosphorus and Preoperative Deep Venous Thrombosis in Geriatric Hip Fracture: A Retrospective Study

**DOI:** 10.3390/diagnostics13030545

**Published:** 2023-02-02

**Authors:** Dong-Xing Lu, Kun Zhang, Teng Ma, Ming Li, Zhong Li, Yi-Bo Xu, Chao-Feng Wang, Cheng Ren, Bin-Fei Zhang

**Affiliations:** 1 Department of Orthopaedic Surgery, Honghui Hospital, Xi’an Jiaotong University, Xi’an 710054, China; 2 Department of Joint Surgery, Honghui Hospital, Xi’an Jiaotong University, Xi’an 710054, China

**Keywords:** hip fracture, serum phosphorus, DVT, complication

## Abstract

Objective: This study aimed to evaluate the association between serum phosphorus level and preoperative deep vein thrombosis (DVT) in geriatric hip fractures. Methods: Older adults with hip fractures were screened between January 2015 and September 2019. Demographic and clinical characteristics of the patients were collected. Multivariate binary logistic regression and generalized additive models were used to identify the linear and nonlinear associations between serum phosphorus levels and preoperative DVT. Analyses were performed using Empower Stats and R software. Results: In this study, 1818 patients were included, with an average age of 79.39 ± 6.87. Of these, 30.25% were males, and 580 patients had DVT. The study found that when serum phosphorus was used as a continuous variable, there was a statistically significant difference in the relationship between blood phosphorus and the occurrence of DVT (*p* < 0.05). Furthermore, we also found curvilinear relationships. Serum phosphorus = 0.71 mmol/L was the inflection point in the curve. When serum phosphorus was <0.71 mmol/L, the serum phosphorus was associated with DVT (OR = 1.64; 95% CI: 1.04–2.59; *p* = 0.0333). With a 0.1 mmol/L increase, the DVT increased 0.64 times. When phosphorus was >0.71 mmol/L, there was no significant difference in the correlation between serum phosphorus levels and DVT (OR = 1.03; 95% CI: 0.98–1.09; *p* = 0.186). Conclusion: Serum phosphorus was nonlinearly associated with preoperative DVT in geriatric patients with hip fractures, and serum phosphorus level could be considered a predictor of DVT risk.

## 1. Introduction

Older adults are prone to serious fractures under low violence due to osteoporosis. As the global population ages, such fractures are set to become a significant healthcare burden. The hip is the stopping point connecting the upper and lower limbs, where stress is concentrated and the first place to land when falling. Therefore, hip fracture is one of the most common types of fractures among older adults and a significant public health problem [1]. By 2030, nearly 2.89 million hip fractures are estimated to occur among individuals aged 65 years and older in the United States and a staggering 6.3 million worldwide by 2050 [2]. When a hip fracture occurs, patients require bed rest for a prolonged period, which causes lower limb muscle atrophy and loss of muscle pump action. The body is in a state of intense stress after a fracture, with activated clotting factors leading to a high risk of blood coagulation. When hip fracture causes edema in areas surrounding the hip, the veins in the region are compressed, and the circumfluent veins choke, further leading to slow blood flow. Therefore, the incidence rate of lower-extremity deep vein thrombosis (DVT) in hip fractures is increased significantly. DVT can block the blood vessels and obstruct venous return, leading to pigmentation of the distal limb, ulceration, skin necrosis, and more serious conditions such as thrombus fall and fatal pulmonary embolism. Therefore, it is crucial to identify relevant indicators for predicting DVT occurrence early.

Phosphorus is an essential element required by the body and is a crucial component of adenosine triphosphate. The cell membrane is composed of a phospholipid bilayer. Therefore, a reduction in blood phosphorus levels changes cell function significantly. Lipids in the platelet membrane play an important role in platelet function, and modification of lipid components can fluidize and harden the environment around the embedded receptor [3]. When blood phosphorus levels are low, platelet function is severely damaged, sharply shortening their life expectancy. Studies have shown that high phosphorus levels can significantly increase serum aldosterone and natriuretic peptide [4]. Aldosterone and natriuretic peptides maintain effective blood volume changes, and aldosterone increases Na^+^ reabsorption in the renal tubules. Natriuretic peptides increase sodium excretion. Therefore, when blood phosphorus levels increase, under the combined action of low aldosterone and high natriuretic peptide, large amounts of Na^+^ and water are discharged, making the blood viscous; thus, the flow rate slows down. Furthermore, when the serum phosphorus level changes, the state of the blood also changes accordingly.

We speculated that the change in serum phosphorus level is related to the occurrence of DVT; however, to the best of our knowledge, no reports published so far support this argument. This study aimed to explore the potential relationship between serum phosphorus changes and DVT in older adults with hip fractures to guide early clinical intervention for thrombosis.

## 2. Materials and Methods

### 2.1. Study Design

In this retrospective cohort study, we recruited older adults who had hip fractures from 1 January 2015 to 30 September 2019 at the largest trauma center in Northwest China.

This retrospective study was approved by the Ethics Committee of the Xi’an Honghui Hospital (No. 202201009). Informed consent was obtained from all patients. All human procedures were performed in accordance with the 1964 Declaration of Helsinki and its later amendments.

### 2.2. Setting

Patients were examined using blood tests to prepare for surgery. Prophylaxis for DVT was initiated on admission. Mechanical thromboprophylaxis (foot intermittent pneumatic compression sleeve, 20 min twice daily) was used to prevent DVT. In patients without contraindications, low-molecular-weight heparin was injected subcutaneously to prevent DVT.

### 2.3. Participants

The demographic and clinical data of the patients were obtained from their original medical records. The inclusion criteria were as follows: (1) age ≥ 65 years; (2) X-ray or computed tomography diagnosis of the femoral neck, intertrochanteric, or subtrochanteric fracture; (3) patients receiving surgical or conservative treatment in the hospital; (4) availability of clinical data in the hospital.

### 2.4. Variables

The variables collected in this study were age, sex, occupation, history of allergy, injury mechanism, fracture classification, hypertension, diabetes, coronary heart disease (CHD), arrhythmia, hemorrhagic stroke, ischemic stroke, cancer, associated injuries, dementia, chronic obstructive pulmonary disease (COPD), hepatitis, gastritis, age-adjusted Charlson comorbidity index (CCI), time from injury to admission, and serum phosphorus. The dependent variable was preoperative DVT, and the independent variable was serum phosphorus level. The other variables were potentially confounding factors.

### 2.5. Data Sources/Measurement

Serum phosphorus levels were measured in electrolyte examination. Doppler ultrasonography was performed to diagnose DVT. The diagnostic criterion was the presence of a constant intraluminal filling defect. All patients underwent ultrasonography of the bilateral lower extremities a day before the scheduled surgery.

### 2.6. Bias

We collected all the patients with serum phosphorus and preoperative DVT results. Because we screened DVT for all the admission patients and examined the blood test, the selection bias was low. As for the measurement bias, we used objective indicators of blood phosphorus and DVT and measured them with uniform standards and methods. The instruments and equipment used were also calibrated to control for measurement bias.

### 2.7. Statistics Analysis

Continuous variables were reported as mean ± standard deviation (Gaussian distribution) or median (skewed distribution), and categorical variables were presented as frequencies and percentages. We used χ^2^ (categorical variables), one-way analysis of variance (ANOVA) (normal distribution), or Kruskal–Wallis H test (skewed distribution) to test for differences among different serum phosphorus levels. Using three distinct models, we used univariate and multivariate binary logistic regression models to test the association between serum phosphorus levels and preoperative DVT. To account for the nonlinear relationship between serum phosphorus and preoperative DVT, we used a generalized additive model (penalized spline method) and smooth curve fitting to address nonlinearity. Additionally, a two-piecewise binary logistic regression model was used to explain nonlinearity further. To test the robustness of our results, we performed a sensitivity analysis. We converted serum phosphorus into a categorical variable according to the quartile and calculated the *p* for the trend to verify the results of serum phosphorus as the continuous variable and to examine the possibility of nonlinearity.

All analyses were performed using statistical software packages R (http://www.R-project.org (accessed on 11 December 2022), R Foundation) and Empower Stats (http://www.empowerstats.com (accessed on 11 December 2022), X&Y Solutions Inc., Boston, MA, USA). Odds ratios (OR) and 95% CI were calculated. Statistical significance was set at a *p*-value < 0.05 (two-sided), which was considered to represent statistical significance.

## 3. Results

### 3.1. Patient Characteristics

From 1 January 2015 to 30 September 2019, 1818 patients with geriatric hip fractures were included in this retrospective cohort study. Among the patients included in this study, 580 (31.90%) had preoperative DVT. The serum phosphorus level was 1.01 ± 0.22 mmol/L. The patients were divided into three groups according to their phosphorus levels. Demographic and clinical characteristics, including comorbidities, factors associated with injuries, and treatment, are shown in Table 1.

### 3.2. Univariate Analysis of the Association between Variates and DVT

Sex, fracture classification, multiple injuries, dementia, and time to the operation were found to be potential confounding factors. According to the analysis of preoperative factors and the occurrence of DVT, confounding factors were determined to have *p* < 0.1, as shown in Table 2.

### 3.3. Multivariate Analysis between Preoperative Serum Phosphorus and DVT

Three models were used to illustrate the correlation between serum phosphorus levels and DVT, as shown in Table 3. When serum phosphorus level was a continuous variable, stable linear regression was observed. The fully-adjusted model showed that the mortality risk decreased by 6% (OR = 1.06; 95% CI: 1.01–1.11; *p* = 0.0221) with every 0.1 mmol/L increase in serum phosphorus level after controlling the confounding factors. When the serum phosphorus was changed to a categorical variable, we found no statistically significant differences upon comparing medium and low serum phosphorus levels in all three models (*p* < 0.05). Additionally, the *p* for the trend also showed an unstable linear correlation in the three models. This reminds us of the possibility of nonlinear correlation.

### 3.4. Curve Fitting and Analysis of Threshold Effect

**Figure 1:**
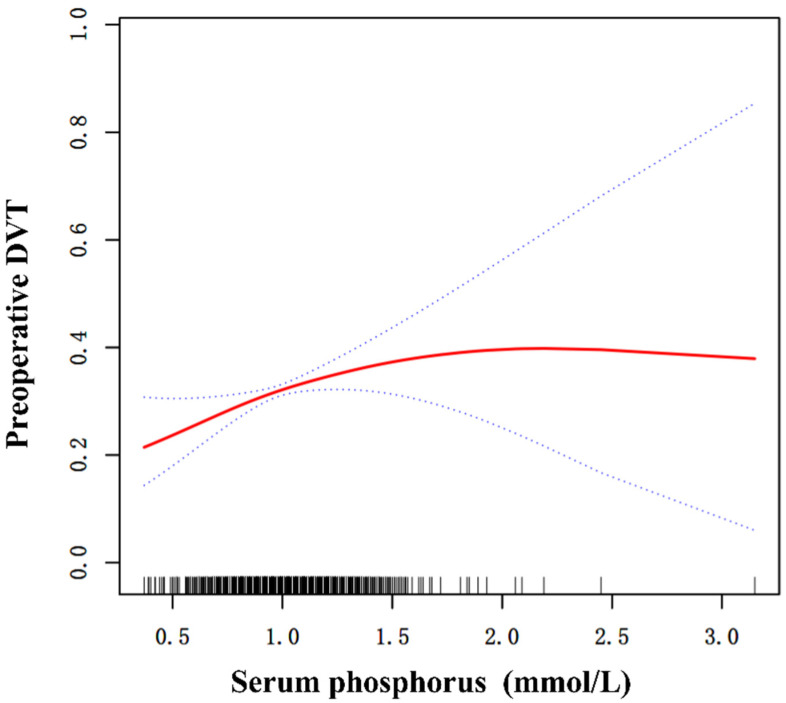
Curve fitting between preoperative serum phosphorus and DVT. Adjusted for sex, fracture classification, multiple injuries, dementia, time to operation.

After curve fitting, we found a possible curvilinear relationship, which was confirmed upon further analysis, as shown in Figure 1. A serum phosphorus level of 0.71 mmol/L was the inflection point in the curve; when the level was less than 0.71 mmol/L, the availability of serum phosphorus was correlated with DVT (OR = 1.64; 95% CI: 1.04–2.59; *p* = 0.0333). In contrast, serum phosphorus was not correlated with DVT when the serum phosphorus level was >0.71 mmol/L (OR = 1.03; 95% CI: 0.98–1.09; *p* = 0.186), as shown in Table 4.

## 4. Discussion

DVT is a common complication of hip fractures among older adults; however, there is currently no particular indicator to predict the occurrence of DVT to guide early anticoagulant therapy. Clinically, many doctors routinely use heparin anticoagulation and Ciccone. Ref. [5] uses bemiparin to significantly affect the prevention of DVT because there are no predictable indicators, so the prevention rate of DVT is still low. Phosphorus (*p*) is a crucial element in bone metabolism. This study explored the relationship between serum phosphorus levels and DVT and found a curved relationship between serum phosphorus levels and DVT. Our findings indicate that the change in serum phosphorus level could be used as an indicator to predict DVT occurrence. When the phosphorus level was less than 0.71 mmol/L, phosphorus was related to DVT, and the preoperative DVT formation rate of patients with low phosphorus levels was lower. With every 0.1 mmol/L increase in phosphorus levels, there was a 0.64-fold increase in the risk of DVT. When the blood phosphorus was close to 0.71 mmol/L, the probability of thrombosis was the highest. When the serum phosphorus level was higher than 0.71 mmol/L, the incidence of DVT did not show a change in serum phosphorus levels.

As an indispensable component of nucleic acids, cell membranes, and adenosine triphosphate, phosphorus plays a crucial biochemical role in regulating the activities of various enzymes and participating in metabolism, both inside and outside the cells [6]. The normal blood phosphorus concentration is 1.1–1.3 mmol/L. Since phospholipids are the primary component of platelets and coagulation factor I, extreme reduction in blood phosphorus severely reduced platelets’ function and life span. This suggests that low concentrations of blood phosphorus will weaken blood clotting ability. Furthermore, phosphorus maintains a stable pH in tissue fluids. Phosphorus is found mainly in bones and teeth in complexes with calcium as hydroxyapatite. Approximately 14% of phosphorus is present in soft tissues as phosphates, and only 1% is present in extracellular fluids as inorganic phosphates, which are used as buffers to regulate mineralization. Parathyroid hormone(PTH) is one of the most important regulatory substances of bone and mineral metabolism, mainly acting on bones, intestines, and kidneys, regulating calcium and phosphorus metabolism [7]. Literature studies have shown that parathyroid hormone can promote oxidative stress of vascular endothelium, activation of the RAAS system, arteriosclerosis, and endothelial dysfunction. Therefore, when blood phosphorus decreases, causing changes in PTH, thereby affecting changes in the blood system, but the connection with DVT needs further study [8,9]. Serum phosphate is only a tiny percentage of total phosphorus; however, it provides crucial insights into body phosphorus levels [10]. Organophosphorus is involved in glycolysis, ammonification, and oxidative phosphorylation, which produce chemical energy by generating adenosine triphosphate through adenosine diphosphate. It also affects the oxygen-carrying capacity of hemoglobin by regulating the synthesis of 2,3 diphosphoglycerate. Phosphorus atoms are also components of DNA, RNA bases, and phospholipids that are involved in cell structure and signaling [11]. Therefore, a reduction in human blood phosphorus levels causes cell damage, tissue hypoxia, and multiple organ damage [8]. When blood phosphorus is close to 0.71 mmol/L, the function of platelets in the blood returns to normal. However, because the concentration is still lower than normal, it can still cause significant damage to the tissues and cells, especially to the vascular endothelium, further leading to aggregation of platelets and coagulation factors. Consequently, special attention should be paid to DVT prevention. Higher blood phosphorus also influences the occurrence of DVT. The human endothelial cells β-glycerophosphate, by increasing the expression of p16 and age-related β-galactose glucoside enzyme activity, induce endothelial cell damage when the blood phosphate levels increase [12,13]. Serum phosphorus levels can improve the level of active oxygen free radicals directly, causing oxidative damage and endothelial cell dysfunction [14]. Endothelial cell injury can cause platelet aggregation and activate the exogenous coagulation system, further accelerating DVT in the lower limbs. High serum phosphorus levels cause insulin resistance, increasing blood glucose levels [13,15,16]. High blood glucose levels increase blood viscosity [17], leading to DVT. Furthermore, higher blood phosphorus is also related to the metabolism of aldosterone, natriuretic peptide, and aldosterone, which can also increase blood viscosity [4]. However, our study found that the occurrence of DVT did not change with the change in blood phosphorus concentration when the blood phosphorus concentration was greater than 0.71 mmol/L. This was possibly because the relationship between the change in blood phosphorus levels and DVT was not examined in this study when the blood phosphorus concentration was greater than 1.3 mmol/L. This aspect requires further clarification in future studies. The detailed mechanism by which serum phosphorus levels affect DVT occurrence of DVT remains unclear. Although the mechanism by which serum phosphorus causes vascular endothelial injury has been studied, corresponding studies have not explained the critical concentration of serum phosphorus that causes vascular endothelial injury. Therefore, future studies need to determine the serum phosphorus concentration that can cause vascular endothelial damage and lead to DVT.

This study adopted multivariate binary logistic regression and generalized additive models to make accurate adjustments for each confounding factor, and the interference of individual factors was excluded. To the best of our knowledge, this is the first study to examine the association between blood phosphorus levels and DVT. In this study, 1818 cases belonging to a large-sample study were collected. However, this was a single-center study, and only cases from our hospital were included. Most patients in our hospital came from Northwest China, and the living conditions and personal physique affected the study, which was limited to older adults with hip fractures. Further research on fractures in diverse populations is needed. Furthermore, this was a retrospective study, even though we controlled the potential selection bias and measurement bias in different ways. The kidneys and the small intestine are the primary organs that maintain phosphorus homeostasis. Most dietary phosphate is absorbed from the gastrointestinal tract and excreted in urine [18]. Once older adults suffer significant trauma, the blood vessels of the gastrointestinal tract and kidney are constricted, leading to different degrees of organ damage and affecting the metabolism of calcium and phosphorus, thus affecting the accuracy of the conclusions [19,20]. The study failed to fully consider the effects of other factors on blood phosphorus, such as COPD, CHD, etc. More prospective studies are needed to study the relationship between phosphorus conversion and DVT alone while controlling for other variables. Since blood phosphorus may change dynamically during follow-up, leading to an underestimation of the correlation of regression dilution bias, the longer the time between exposure assessment and disease, the more pronounced this problem is. In future studies, we need to repeat the blood phosphorus concentration in the investigators to dynamically show the effect of blood phosphorus changes on DVT. More experimental results require further basic experiments to verify. Finally, our findings are only intended to be helpful to clinicians in predicting DVT and cannot be used as an indicator to evaluate the occurrence of DVT.

## 5. Conclusions

This study found that serum phosphorus was nonlinearly associated with preoperative DVT in geriatric patients with hip fractures, and serum phosphorus could be considered a predictor of the risk of DVT. More prospective studies are needed to further investigate the correlation between blood phosphorus and DVT while controlling for confounding variables.

## Figures and Tables

**Table 1:** The demographic and clinical characteristics.

Phosphorus Tertiles	Low	Middle	High	*p*-Value	*p*-Value *
*N*	611	632	575		
Phosphorus (mmol/L)	0.78 ± 0.11	1.00 ± 0.05	1.25 ± 0.17	<0.001	<0.001
Age (year)	79.42 ± 6.68	79.58 ± 7.07	79.14 ± 6.86	0.541	0.598
Sex				<0.001	-
Male	273 (44.68%)	167 (26.42%)	110 (19.13%)		
Female	338 (55.32%)	465 (73.58%)	465 (80.87%)		
Injury mechanism				0.111	-
Falling	587 (96.07%)	613 (96.99%)	555 (96.52%)		
Accident	21 (3.44%)	17 (2.69%)	12 (2.09%)		
Other	3 (0.49%)	2 (0.32%)	8 (1.39%)		
Fracture classification				0.038	-
Intertrochanteric fracture	348 (56.96%)	373 (59.02%)	367 (63.83%)		
Femoral neck fracture	256 (41.90%)	243 (38.45%)	199 (34.61%)		
Subtrochanteric fracture	7 (1.15%)	16 (2.53%)	9 (1.57%)		
Hypertension	309 (50.57%)	316 (50.00%)	292 (50.78%)	0.961	-
Diabetes	112 (18.33%)	123 (19.46%)	129 (22.43%)	0.192	-
CHD	316 (51.72%)	326 (51.58%)	294 (51.13%)	0.978	-
Arrhythmia	195 (31.91%)	201 (31.80%)	178 (30.96%)	0.928	-
Hemorrhagic stroke	6 (0.98%)	15 (2.37%)	10 (1.74%)	0.166	-
Ischemic stroke	204 (33.39%)	211 (33.39%)	159 (27.65%)	0.05	-
Cancer	15 (2.45%)	20 (3.16%)	14 (2.43%)	0.666	-
Multiple injuries	49 (8.02%)	39 (6.17%)	43 (7.48%)	0.431	-
Dementia	21 (3.44%)	29 (4.59%)	22 (3.83%)	0.57	-
COPD	49 (8.02%)	28 (4.43%)	27 (4.70%)	0.011	-
Hepatitis	12 (1.96%)	21 (3.32%)	20 (3.48%)	0.227	-
Gastritis	5 (0.82%)	13 (2.06%)	8 (1.39%)	0.184	-
DVT	173 (28.31%)	205 (32.44%)	202 (35.13%)	0.04	-
aCCI	4.25 ± 1.12	4.23 ± 1.10	4.16 ± 1.10	0.308	0.361
Time to operation (d)	4.09 ± 2.30	4.23 ± 2.57	4.26 ± 2.44	0.466	0.5
Time to admission (h)	53.79 ± 140.18	85.71 ± 316.13	111.62 ± 274.87	<0.001	<0.001

Mean + SD/*N*(%)*. p*-value *: For continuous variables, we used the Kruskal–Wallis rank-sum test and Fisher’s exact probability test for count variables with a theoretical number of <10.

**Table 2:** Effects of factors on DVT measured by univariate analysis.

	Statistics	OR (95% CI)	*p* Value
Age (year)	79.39 ± 6.87	1.00 (0.99, 1.02)	0.5892
Sex			
Male	550 (30.25%)	1	
Female	1268 (69.75%)	1.25 (1.01, 1.56)	0.0433
Injury mechanism			
Falling	1755 (96.53%)	1	
Accident	50 (2.75%)	1.11 (0.61, 2.01)	0.7284
Other	13 (0.72%)	2.52 (0.84, 7.52)	0.0987
Fracture classification			
Intertrochanteric fracture	1088 (59.85%)	1	
Femoral neck fracture	698 (38.39%)	0.67 (0.55, 0.83)	0.0002
Subtrochanteric fracture	32 (1.76%)	1.87 (0.93, 3.78)	0.0812
Hypertension	917 (50.44%)	1.12 (0.92, 1.37)	0.2494
Diabetes	364 (20.02%)	0.92 (0.72, 1.18)	0.5192
CHD	936 (51.49%)	1.08 (0.88, 1.31)	0.4571
Arrhythmia	574 (31.57%)	1.06 (0.86, 1.31)	0.5976
Hemorrhagic stroke	31 (1.71%)	1.55 (0.76, 3.19)	0.2303
Ischemic stroke	574 (31.57%)	0.86 (0.69, 1.06)	0.1556
Cancer	49 (2.70%)	1.14 (0.63, 2.07)	0.6711
Multiple injuries	131 (7.21%)	1.55 (1.08, 2.22)	0.0183
Dementia	72 (3.96%)	1.65 (1.02, 2.66)	0.0401
COPD	104 (5.72%)	0.90 (0.58, 1.39)	0.6369
Hepatitis	53 (2.92%)	0.69 (0.36, 1.29)	0.245
Gastritis	26 (1.43%)	0.50 (0.19, 1.34)	0.1706
aCCI	4.22 ± 1.11	1.00 (0.91, 1.09)	0.9615
Time to operation (d)	4.19 ± 2.44	1.05 (1.01, 1.10)	0.0113
Time to admission (h)	83.18 ± 256.36	1.00 (1.00, 1.00)	0.3094
Phosphorus (×0.1 mmol/L)	10.08 ± 2.23	1.06 (1.02, 1.11)	0.0059

**Table 3:** Univariate and multivariate results by linear regression.

Exposure	Non-Adjusted Model	Minimally-Adjusted Model	Fully-Adjusted Model
Phosphorus (×0.1 mmol/L)	1.06 (1.02, 1.11) 0.0059	1.06 (1.01, 1.10) 0.0170	1.06 (1.01, 1.11) 0.0221
Phosphorus tertiles			
Low	1	1	1
Middle	1.22 (0.95, 1.55) 0.1144	1.18 (0.92, 1.51) 0.1891	1.14 (0.88, 1.48) 0.3115
High	1.37 (1.07, 1.75) 0.0118	1.31 (1.02, 1.69) 0.0336	1.30 (1.00, 1.69) 0.0542
*p* for trend	0.0117	0.0339	0.0542

Data in table: OR (95% CI) *p*-value; Outcome variable: DVT; Exposed variables: serum phosphorus; Minimally adjusted model was adjusted for sex. The fully adjusted model was adjusted for sex, fracture classification, multiple injuries, dementia, and time to operation.

**Table 4:** Nonlinearity of preoperative serum phosphorus and DVT.

Outcome	OR (95% CI) *p*-Value
Fitting model by stand linear regression	1.06 (1.01, 1.11) 0.0221
Fitting model by two-piecewise linear regression	
Inflection point	0.71 mmol/L
<0.71 mmol/L	1.64 (1.04, 2.59) 0.0333
>0.71 mmol/L	1.03 (0.98, 1.09) 0.1860
*p* for log-likelihood ratio test	0.036

Adjusted for age, sex, fracture classification, multiple injuries, dementia, time to operation.

## Data Availability

The data were provided by Xi’an Honghui Hospital. According to relevant regulations, the data cannot be shared but could be requested from the corresponding author.
